# Mouse Acetylcholinesterase Enhances Neurite Outgrowth of Rat R28 Cells Through Interaction With Laminin-1

**DOI:** 10.1371/journal.pone.0036683

**Published:** 2012-05-03

**Authors:** Laura E. Sperling, Janine Klaczinski, Corina Schütz, Lydia Rudolph, Paul G. Layer

**Affiliations:** Entwicklungsbiologie und Neurogenetik, Fachbereich Biologie, Technische Universität Darmstadt, Darmstadt, Germany; Aix Marseille University, France

## Abstract

The enzyme acetylcholinesterase (AChE) terminates synaptic transmission at cholinergic synapses by hydrolyzing the neurotransmitter acetylcholine, but can also exert ‘non-classical’, morpho-regulatory effects on developing neurons such as stimulation of neurite outgrowth. Here, we investigated the role of AChE binding to laminin-1 on the regulation of neurite outgrowth by using cell culture, immunocytochemistry, and molecular biological approaches. To explore the role of AChE, we examined fiber growth of cells overexpressing different forms of AChE, and/or during their growth on laminin-1. A significant increase of neuritic growth as compared with controls was observed for neurons over-expressing AChE. Accordingly, addition of globular AChE to the medium increased total length of neurites. Co-transfection with PRIMA, a membrane anchor of AChE, led to an increase in fiber length similar to AChE overexpressing cells. Transfection with an AChE mutant that leads to the retention of AChE within cells had no stimulatory effect on neurite length. Noticeably, the longest neurites were produced by neurons overexpressing AChE and growing on laminin-1, suggesting that the AChE/laminin interaction is involved in regulating neurite outgrowth. Our findings demonstrate that binding of AChE to laminin-1 alters AChE activity and leads to increased neurite growth in culture. A possible mechanism of the AChE effect on neurite outgrowth is proposed due to the interaction of AChE with laminin-1.

## Introduction

Acetylcholinesterase (AChE) is the enzyme that terminates neurotransmission at cholinergic synapses in central and peripheral nervous systems. Several other potential functions have been associated to AChE, as for example stimulation of neurite outgrowth, adhesion, regulation of cell differentiation, apoptosis, hematopoiesis and thrombopoiesis [1]–[8]. Most prominent among the morphogenic functions is facilitation of neurite growth. There are many documented examples where neurite growth is preceded by or associated with cholinesterase expression, occurring long before the onset of cholinergic neurotransmission [9], [10], as shown by our laboratory [5] and others [11]–[14]. Various mechanisms have been proposed for this function of AChE. One of them would be that expression of the enzyme during development may regulate the levels of acetylcholine (ACh), establishing permissive pathways for the axonal elongation. However, the increased neurite growth cannot be, or not only, the result of the esteratic activity, since at least one compound was found that inhibits AChE activity but not neurite outgrowth [5], [15]. Also indicating a non-catalytic mechanism, treatment of cell cultures with an anti-AChE monoclonal antibody, which did not affect AChE activity, led to a detachment of neurites [16]. Noticeably, forms of AChE that hydrolyzed ACh, but lacked the C-terminal domain, failed to enhance neurite growth, again demonstrating the independence of the catalytic and neuritogenic activity from each other [1].These *in vitro* findings were complemented by *in vivo* results from an AChE knock-out mouse, where formation of neural networks in the inner retina was distorted [17]. However, transgenic mice overexpressing the human synaptic AChE in central cholinergic neurons exhibited diminished dendritic branching and reduced numbers of spines in cortical neurons [18].

To explain this on a structural basis, AChE is highly homologous to a class of cell adhesion molecules named ‘cholinesterase-like cell adhesion molecules’ [19], [20]. Moreover, AChE is also able to interact with other proteins [21]–[23]; e.g. its interaction with laminin-beta 1 [23] supports the hypothesis that AChE can exert cell adhesion properties. Therefore, we propose that AChE can act morphogenically through its binding to laminin-1. An outgrowth promoting activity of laminin-1 has been established for many neuronal cells and cell lines, acting in the nanomolar range [24]–[27], clearly reflecting an important function during neuronal development *in vivo*. Laminin occurs in at least eleven isoforms [28], some of which are expressed in developing axon tracts in a spatio-temporally order, further supporting its significance for the developing brain.

The neurite growth promoting capability of AChE was found on various molecular forms of AChE. However, the precise site on AChE responsible for the neuritogenic activity has not yet been clearly defined. In general, the non-catalytic structural functions of AChE were mainly attributed to a few distinct sites on AChE, e.g., the peripheral anionic site (PAS), the C-terminal tail (t)-peptide of the synaptic form of AChE [29], or, the C-terminal ARP peptide of the stress-associated AChE-R form. The PAS lies at the entrance of the active site gorge of AChE, and it is probably involved in protein-protein interactions [4], [23], [30]–[34], as well as in cell-substrate adhesion [32], [35], including deposition of beta-amyloid in Alzheimer's disease [36], [37]. The C-terminal t-peptide appears to increase apoptosis [38] and is interacting with ColQ and PRiMA [39]. The ARP peptide promotes neuronal development and plasticity [40].

To further clarify which form of AChE influences neurite growth, and to determine the significance of the AChE/laminin-1 interaction for neurite outgrowth, we reconstituted the interaction *in vitro* using the R28 neuronal cell line [41] by over-expressing the synaptic AChE and cultivating these cells on laminin-1-coated culture dishes. The following questions were addressed: 1) does binding of AChE to laminin-1 have a neurite growth promoting function; and 2) which variant of AChE (secreted or membrane-bound) promotes process extension by binding to laminin-1. This study demonstrates a direct correlation between AChE expression and neurite outgrowth; the membrane-anchored form seems to have the strongest effect on neurite outgrowth when compared with the soluble extracellular form. We also consistently show that AChE and laminin-1 in combination more than additively increased neurite growth.

## Results

We analyzed the efficacy of promoting neurite outgrowth of three different AChE forms: the tetrameric secreted AChE form (E6-AChE or S-AChE), the PRiMA membrane-anchored S-AChE form and the R395C-AChE mutant, which is retained within the cell, therefore not being available for the interaction with laminin-1. A series of controls was used, including cells treated only with the transfection reagent, cells transfected with the empty vector and GFP-overexpressing cells.

### Generation of stably transfected cells and analysis of AChE enzymatic activity in different cellular compartments in the absence or presence of laminin-1

**Figure 1:**
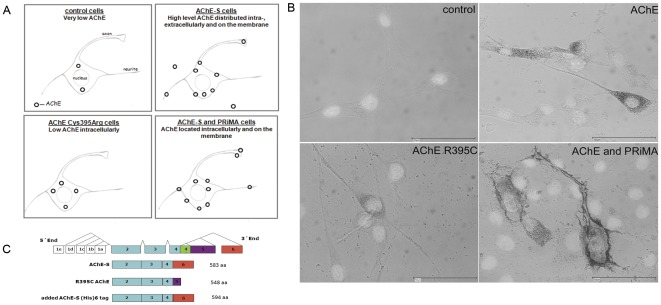
Distribution of AChE activity in transfected R28 cells. (**A**) Cartoon showing the distribution of AChE based on activity stainings. (**B**) Distribution of AChE activity in the cell as shown by Karnovsky and Roots staining. AChE activity was detected by enzymatic staining on paraformaldehyde fixed cells after 24 hours in culture. Representative images of control R28 cells and cells expressing wild type AChE, the 395Arg to Cys mutation and PRiMA. Pictures show merged view of Karnovsky Roots (brown) and the cell nucleus (DAPI). Control cells show no staining (upper left); wild type AChE staining is diffusely distributed in the cytoplasm and punctate along neurites and on the plasma membrane (upper right); staining intensity of mutated AChE is weaker when compared with wild type and is not localized on the plasma membrane (left). Scale bar, 50 µm. (**C**) Scheme of the transfected plasmids and plasmids used for expression of recombinant AChE.

To explore a structural role of AChE in neurite outgrowth involving its binding to laminin-1, we generated different cell lines overexpressing AChE. First, the rat retinal R28 cell line was stably transfected with plasmids encoding either the exon 6 containing form of AChE (E6-AChE), the R395C AChE-548 mutant, GFP or the empty vector (Fig. 1C). However, overexpression of E6-AChE in R28 cells will lead to both membrane-bound and secreted AChE. Therefore, transient transfections with PRiMA of the R28-E6-AChE stably expressing cells were performed, in order to achieve increased anchoring of the enzyme to the cell membrane and avoid secretion (see Fig. 1 for overview). Additionally, R395C AChE mutant overexpressing cells were obtained. These cells served as a negative control of the binding to laminin-1, since mutant AChE is not secreted and therefore not able to interact with laminin-1. The best G418-resistant clones were selected by screening for increased AChE activity or green fluorescence (transfection efficiency was: 33% E6-AChE positive cells, 81% GFP positive cells and 5% R395C AChE positive cells).

**Figure 2:**
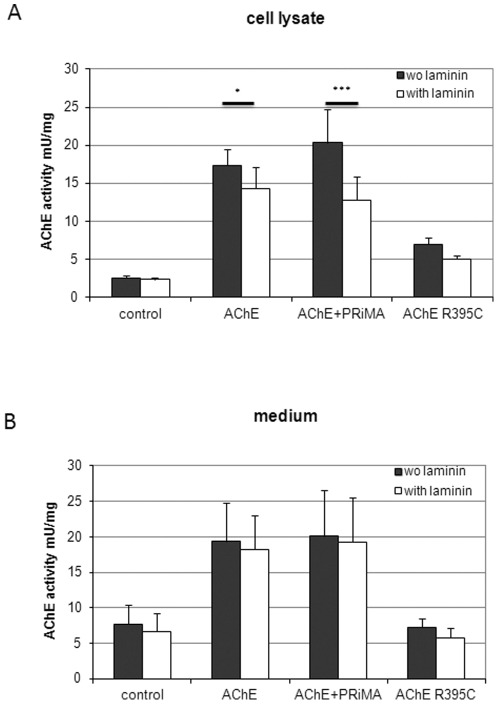
Secreted and cell-associated AChE activity in control and transfected cells. Increase of AChE activities following AChE-transfection is diminished by cultivation on laminin-1, in particular so if the PRiMA anchor is co-transfected. R28 cells were transfected with E6-AChE, R395C AChE, PRiMA and GFP, and AChE activity in cell lysates (**A**) and medium (**B**) was determined. Control clones produced by transfecting with empty vector or GFP showed activity levels similar to those of untransfected cells. Therefore, the GFP expressing cell line was used as control for further experiments. Results are given as means ± standard deviation for at least five separate experiments. * p<0.05; *** p<0.001. All activities were significant increased when compared with control cells.

AChE enzymatic activity measurements in cell lysates and media of E6-AChE, R395C AChE-transfected and control cells revealed activity in all probes (Fig. 2). Control cells express moderate amounts of AChE, which is mostly secreted (Fig. 2B). The cell-associated activity of E6-AChE overexpressing cells was 6.7-fold higher than of control-GFP cells. As expected, the R395C-AChE cells show much lower activity than E6-AChE, with only 2.7-fold increase over control cells (see Fig. 2A) and secrete no AChE when compared to control (Fig. 2B). Predictably, the cell-associated activity of PRiMA-transfected E6-AChE cells was highest (Fig. 2A). On the other hand, the secreted activity of these cells remained comparable to that of E6-AChE cells, which can be explained by low efficiency of the transient transfection with PRiMA.

Interestingly, all cells cultivated on laminin-1 showed reduced AChE activity when compared to cultures without laminin-1, suggesting that the interaction might affect the catalytic site of the enzyme. Noticeably, a most pronounced difference was observed in the case of PRiMA-AChE cell associated activity (Fig. 2A) indicating that the membrane-bound AChE is the AChE form that interacts with laminin-1. Additional measurements pointed to the fact that culture on laminin-1 influences AChE, but not butyrylcholinesterase (BChE) activity (not shown).

### Cellular distribution of AChE in control and transfected cells

Since the AChE localisation at the cell surface is important for the physical interaction with laminin-1, we first investigated AChE distribution using the Karnovsky and Roots histochemical staining (Fig. 1B). Cells were fixed with paraformaldehyde and stained for AChE activity and DAPI to facilitate microscopy of unstained cells. Control cells did not show any staining for the incubation time used (∼1.5 hours). In E6-AChE cells the activity is evenly distributed over the entire cell body, being high in the perinuclear region with small patches on the axon and neurites (Fig. 1B, up right). Transfection of E6-AChE overexpressing cells with PRiMA leads to a diffuse intracellular staining and high concentration of the activity on the cell membrane (Fig. 1B, down left). Here is to note also the modification of membrane appearance with the formation of numerous sprouting spike-like extensions (Fig. 1 and Fig. 6). Intracellular AChE was present throughout the soma and neurites, surface AChE was selectively found on growth cones and discrete patches along neurites, including at many branch points. AChE R395C cells show an AChE distribution similar to E6-AChE cells, but the staining was much weaker, while the incubation time was increased to up to 8 hours.

### Cell morphology is altered as a result of AChE overexpression and/or culture on laminin-1

**Figure 3:**
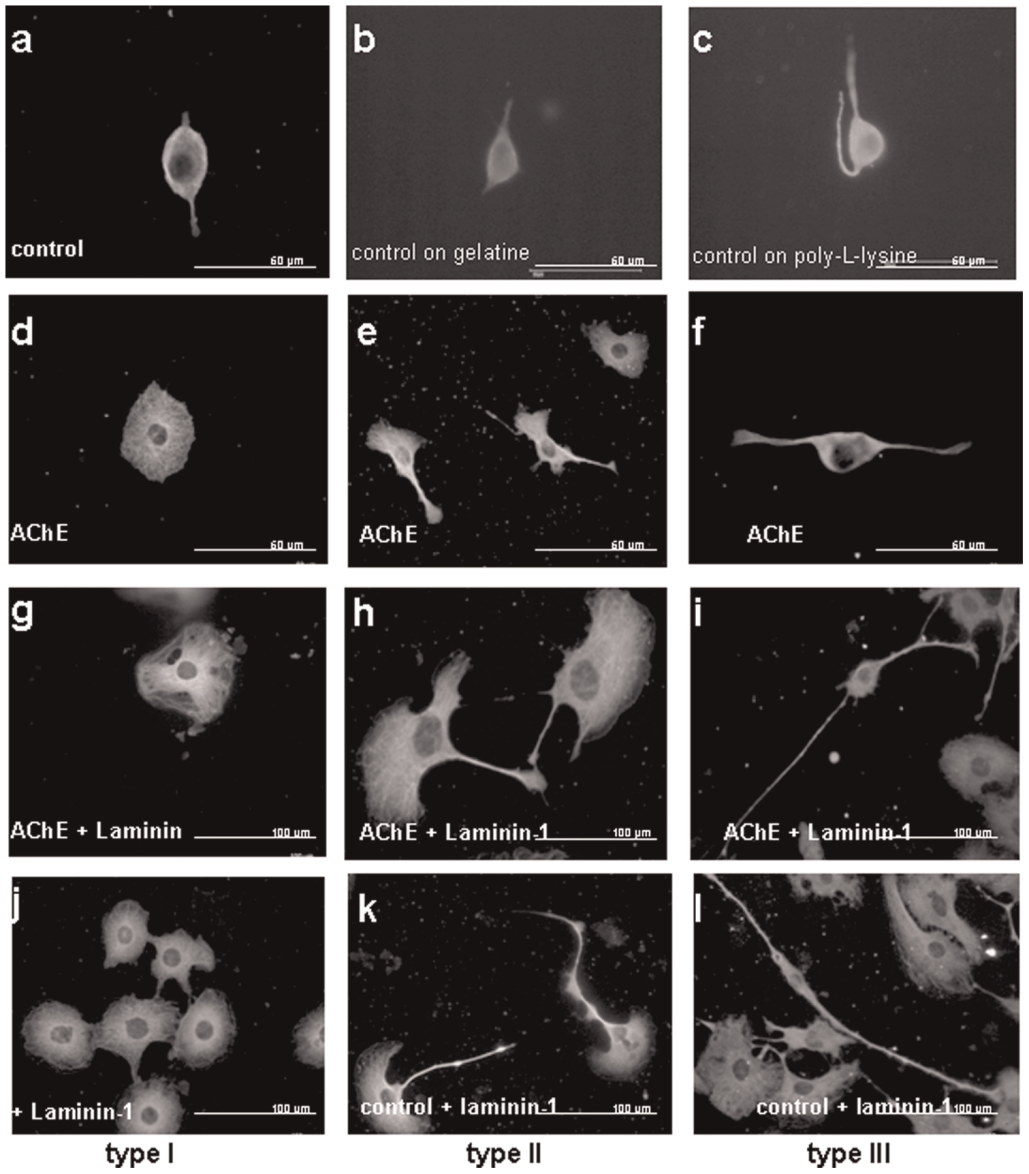
Altered neurite lengths and cell morphology as a result of AChE overexpression or/and culture on laminin-1. Pictures show immunostaining with an anti-α tubulin antibody. Low density culturing of cells led to the formation of three distinct morphologies, arbitrarily named type I, II and III. Type I is characterized by the absence of neurites and a round cell body (A, D, G. J), type II has 1–2 neurites (B, E, H, K) and type III resembles presents a bipolar neuronal morphology (C, F, I, L). Cultivation of cells on gelatine or poly-L-lysine coated surface had no effect on cell morphology. Note that the cells on laminin-1 and AChE overexpressing cells on laminin-1 are bigger than AChE overexpressing cells only. Scale bar (A–F) 50 µm, (G–L) 100 µm.

**Figure 4:**
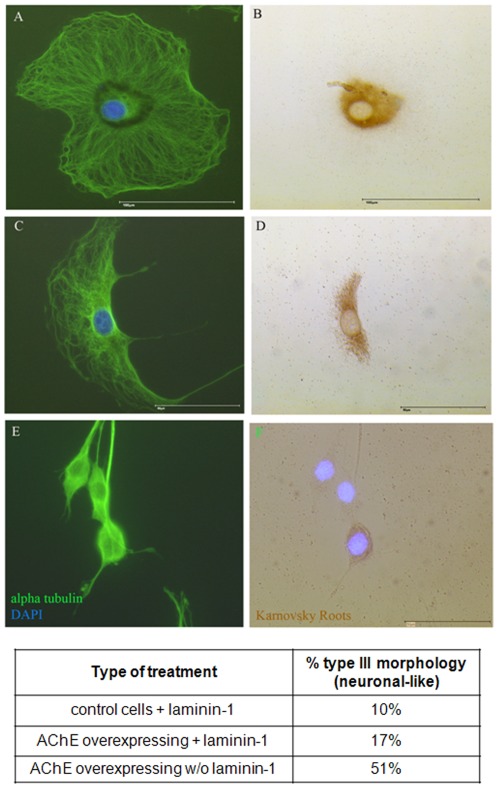
Intracellular distribution of AChE activity in the 3 types of cellular morphology. Cell morphology, particularly so neurite lengths are altered as a result of AChE overexpression and culture on laminin-1. Pictures show merged view of Karnovsky Roots (brown), immunostaining with an anti-alpha tubulin antibody (green) and cell nuclei stained with DAPI (blue). Scale bar 50 µm. Table: AChE overexpression stimulates the formation of type III morphology (neuronal-like). Laminin-1 leads to a strong shift to the type I and type II cells.

Noticeably, as soon as the cells were overexpressing AChE, or were cultivated on laminin-1, the cell morphology changed. As shown in Figure 3, the morphology of AChE-transfected or/and on laminin-1 cultivated cells is noticeably different from the parental cell line. Typically, under AChE over-expression three different morphologies appear, arbitrarily called type I, II and III. Type I shows no neurites and a much increased cell surface (Fig. 3D, G and H), type II shows also an increased cell body with up to 2 neurites (Fig. 3E, H and K), the bipolar morphology of type III cells is similar to that of control cells (Fig. 3F, I and L). Interestingly, AChE overexpression led preferentially to the formation of type III cells (neuronal-like), while laminin-1 culturing led to formation of type I cells (see Table Fig. 4). Double staining of the tubulin cytoskeleton and AChE activity showed the intracellular localization of AChE in these three cell types (Fig. 4). In type I and II cells, AChE is localized perinuclearly (Fig. 3A–D). Type III cells show AChE activity distributed on soma and neurites (Fig. 4E, F). While type I cells have no neurites and type II cells express AChE only perinuclearly, only type III cells were chosen for the neurite length measurements.

### Effects of expressing the synaptic form of AChE on neurite growth – The E6-derived C-terminus is essential for growth promotion

**Figure 5:**
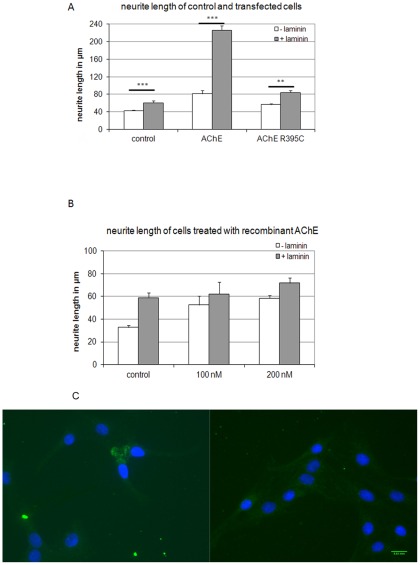
Measurements of neurite length in control and with AChE transfected or treated cells. (**A**) Neurites of type III cells (neuron-like) are longer in AChE-transfected R28 than in control cells (open bars), and even much longer when they are grown on laminin-1 (dark bars). Note drastic increase of neurite lengths in AChE-overexpressing cells grown on laminin-1 (upper, right). (**B**) Supplementation of cultures with recombinant AChE leads to similar results; note, however increase in presence of AChE is more pronounced than in AChE-overexpressing cells (cf. upper). Neurites of α-tubulin-labelled cells were measured using specific software. At least 100 cells from three different passages were measured and averaged. In the case of stably transfected clones, only AChE-positive cells were included in the measurements. Values are mean ± SEM from 5 different experiments, *p<0.05, **p<0.01, ***p<0.001. (**C**) Immunostaining with an anti (His)_6_ Tag antibody (green) of R28 cells treated with (His)_6_ Tag E6-AChE. Cell nuclei are stained with DAPI (blue). Scale bar 200 µm.

We next evaluated the neurite growth of various cell-substrate combinations. As mentioned above, only neurites of type III cells were measured. Cells were cultured alternatively either on plastic or on laminin-1 coated dishes. As expected, R28 cells overexpressing E6-AChE had longer neurites than control cells (Fig. 5A). Laminin-1 had a strong and significant neurite length-increasing effect, which was potentiated by overexpression of E6-AChE. AChE overexpressing cells cultured on laminin-1 showed the longest neurites (Fig. 5A). To rule out the fact that the strong effect of AChE and laminin-1 on neurite growth was not only additive, but due to AChE-laminin-1 interactions, a series of different types of controls was run in parallel.

### Effects of the intracellular AChE retention on neurite growth

An R395C AChE mutant was overexpressed in R28 cells and employed as a further control to establish the relevance of AChE-laminin-1 interaction for neurite outgrowth. These cells showed lower transfection efficiency, fact that is probably due to our detection limits or to the fact that the mutant protein is rapidly degraded. For estimating the transfection efficiency we counted the cells that show AChE activity. However, the R395C AChE mutant shows only about 13% enzymatic activity when compared to wild type AChE [42], a fact that makes the detection of the transfected cells more difficult. R395C AChE transfected cells do not undergo apoptosis (results not shown). Retention of AChE in the cell led to a slight increase in neurite growth, as well as culturing on laminin-1 of these cells. However, the R28 cells stably expressing the AChE mutant form grown on laminin-1 did not show significantly longer neurites as control cells (Fig. 5A, dark bars).

### Addition of soluble AChE in culture

First, different concentrations of recombinant mouse AChE were added to the culture medium of control cells (Fig. 5B). An increase in neurite growth was observed, an increase that was not concentration-dependent. Importantly, no significant neurite length increase was observed on cells treated with AChE and cultured on laminin-1 when compared with single treatments. This supports the idea that the effect is related to a cell-ECM interaction. Since soluble AChE was reported by others [43] to have robust effects on neurite growth, we tested whether the added enzyme penetrated the cell (Fig. 5C). Only in about 0.5% of cells we could detect cell-associated recombinant AChE, mostly to the cell body (Fig. 5C). This may be the reason why we see no significant increase in neurite length.

### PRiMA overexpression in a stably AChE-transfected R28 cell **line** localizes AChE to cell membrane and changes cell morphology

**Figure 6:**
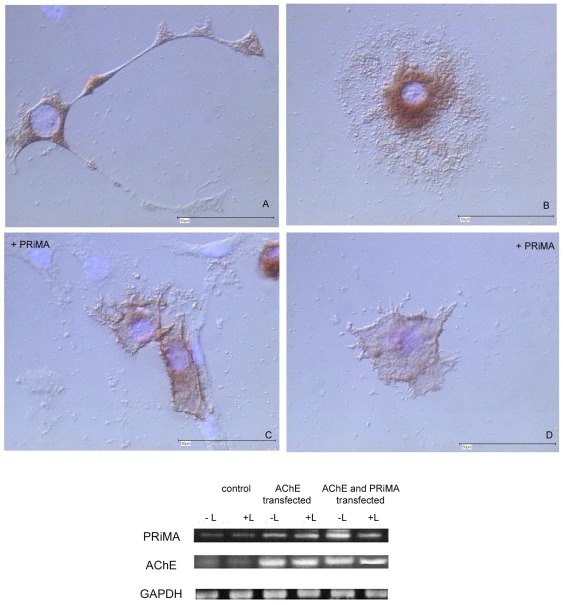
PRiMA overexpression in a stably AChE-transfected R28 cell line **localizes AChE to cell membrane and changes cell morphology**. Note strong AChE activity on membrane of PRiMA transfected cells, with multiple emanating short side processes (C, D), as compared with more diffuse, but still localized AChE expression in control cells (A, B). (A–D) Karnovsky-Roots staining; all cells were grown on laminin-1. Scale bar 50 µm. Fig. 6 (lower). Expression of PRiMA is strongly increased in cells overexpressing AChE, and more so in AChE plus PRiMA co-transfected cells, as detected by RT-PCR. Total RNA of control, of AChE transfected, and of AChE plus PRiMA transfected cells was used for the analyses. Note: GAPDH primers were used as internal control.

R28 cells transfected with E6-AChE show homogeneous distribution of AChE throughout the cell body and neurites. To ensure that most of the AChE is membrane anchored and therefore able to interact with laminin-1, we employed PRiMA transient transfections of R28 E6-AChE overexpressing cells. Due to their CNS origin, it was likely that the cells express the neuronal AChE-anchor PRiMA. We performed RT-PCR on total RNA isolated from R28 cells and investigated the expression of PRiMA and AChE. The experiment was repeated several times and a representative gel is shown in Figure 6 (lower). Control cells constitutively express PRiMA; AChE expression is barely detectable. The transfection with E6-AChE leads to a strong increase in the PRiMA transcripts, suggesting a regulation of PRiMA transcript level by AChE levels. Laminin-1 did not affect the amount of AChE and PRiMA mRNA, although the AChE activity of cells on laminin-1 is significantly reduced.

**Figure 7:**
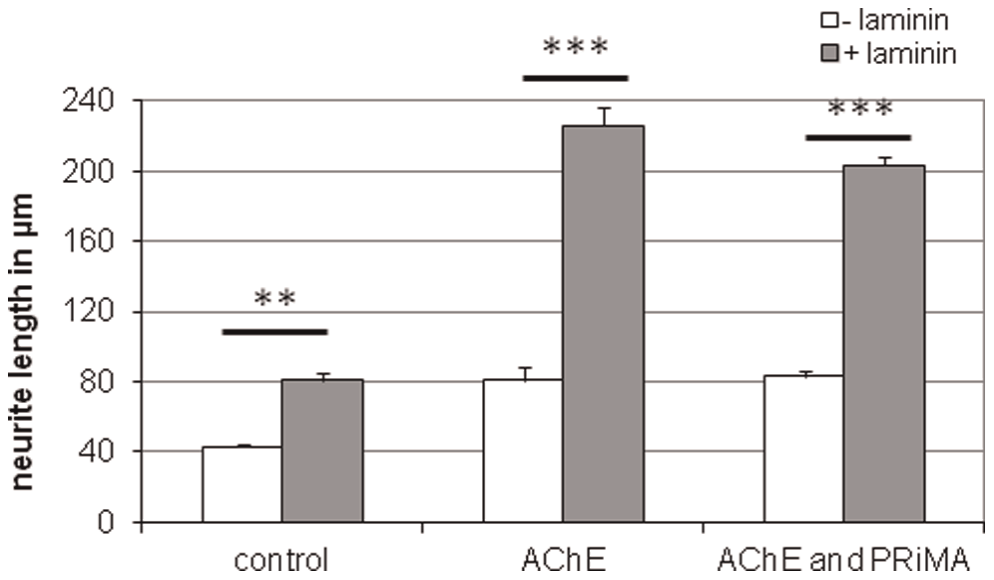
Comparison of neurite length of AChE and AChE+PRiMA overexpressing cells grown in presence (dark bars) or absence (white bars) of laminin-1. Note that there are no significant differences in neurite length of AChE and AChE+PRiMA overexpressing cells.

A Karnovsky and Roots staining was used to investigate the distribution of AChE in PRiMA overexpressing cells. As expected, most of the AChE appears located to the cell membrane, sometimes in a patch-like distribution. But most strikingly was the fact that these cells undergo morphological changes (Fig. 6C, D). Figure 6 shows AChE + PRiMA-overexpressing cells (C and D) that present numerous dendrites sprouting from multiple membrane sites. Neurite lengths of PRiMA- and AChE-overexpressing cells were measured in the presence or absence of laminin-1 and compared with the control and E6-AChE overexpressing cells (Fig. 7). No significant differences between neurite length of AChE on laminin-1 and AChE and PRiMA on laminin-1 cells were observed.

Several trends emerged from these experiments. Either the overexpression of AChE, or the treatment with laminin-1 led to the formation of the same three distinct morphological cell types. While AChE seems to promote neurite growth both in membrane bound and soluble form, culturing on laminin-1 also promotes neurite growth of R28 cells. Retention of AChE in the intracellular compartments had no significant effect on neurite length; addition of recombinant AChE to the culture medium had a slight increasing effect of neurite growth. PRiMA overexpression led to a solid localization of AChE to the cell membrane and to a membrane budding (sprouting). However, the effects on neurite growth were comparable with the effects of E6-AChE overexpression. The culture on laminin-1 of the E6-AChE expressing cells led to a solid and strong increase in fiber growth.

## Discussion

We and others have previously shown that AChE is able to bind to laminin-1 [23], [31]. Here we could demonstrate that the interaction between AChE and laminin-1 might have a role in neurite growth. Numerous studies have documented AChE's ability to promote neurite outgrowth, using both addition of AChE to cell cultures and transfection of cells with AChE cDNA [12], [25], [44], [45]. AChE may exert this effect by three ways, by using catalytic or non-catalytic mechanisms, or a combination of both. This paper suggests that neurite growth promoting functions can be explained by a non-catalytic, adhesive mechanism, through an interaction with the extracellular matrix protein laminin-1.

### AChE and laminin-1 change cell morphologies

A first interesting finding of this study is the fact that both AChE and laminin-1 lead to similar morphological changes of R28 cells. These changes were not seen by transfection with other genes (results from our laboratory, not shown) or by cultivating the cells on other substrates.

One cell type generated by the treatment shows no neurites, but a large cell body, a second cell type has also a large cell body, but presents neurites and a third cell type resembles the neuronal morphology with a small body and two long neurites. That implies that AChE and laminin-1 in different cell types lead to different size/shape/neurite building patterns.

R28 cells are proliferative retinal progenitor cells that express neuronal characteristics [46]. Immunocytochemical results [46] illustrate that despite the clonal (single cell) origin of R28 cells, they are a heterogeneous population which most likely cannot be further purified by additional serial dilutions. A heterogeneous population derived from a single cell supports the concept of R28 cells as retinal precursor cells. In addition, double-immunolabelling has identified dual glial-neuronal marker expression within individual R28 cells. This cellular heterogeneity might explain why overexpression of AChE or growth on laminin-1 leads to the appearance of different cellular morphologies. R28 are neural precursor cells; in this study these cells are overexpressing proteins that have roles in neurite growth and adhesion, hallmarks of neuronal differentiation; therefore the morphological changes observed can be linked to the differentiation process. Further studies are necessary in order to see whether changes in expression patterns of neural-precursor/neuronal/glia markers accompany these morphological changes, implying that different morphologies reflect different cell types. It is also possible that these distinct morphologies do not reflect different cell types but different time-frames during the differentiation of a single cell type. Similar morphologies were observed during neuritogenesis of cortical neurons [47].

Intriguing is the fact that two different molecules alone and in combination lead to formation of same morphological types. This can be a hint that these two molecules use the same signaling mechanism, probably linked to cytoskeletal changes. The cytoskeleton plays a fundamental role and is instrumental for the reorganization of morphological structures during neurite growth. The process of forming of neurites implies F-actin and microtubule dynamics. Connections between the cytoskeleton and cholinergic components were proposed by others [48]. Woolf proposed that acetylcholine may direct consciousness through a cascade of effects leading to a momentary phosphorylation of MAP-2 that interrupts the binding of MAP-2 to microtubules and promotes a process such as microtubular coherence [48]. Besides microtubuli, another candidate would be the actin cytoskeleton, since events as growth cone motility are mediated by changes in actin dynamics. It was documented that the laminin receptor connectin is linked to the actin cytoskeleton [49].

Not only laminin-1 is connected to the cytoskeleton, but also AChE seems to have morphoregulatory effects on neurons, fibroblasts and astrocytes [50]. Fibroblasts and astrocytes express AChE with little catalytic activity that is functionally important for polarized migration. The distribution of AChE at sites of membrane protrusion in neurons, fibroblasts and astrocytes reflects this role. In cortical neurons AChE is in the motile growth cones and neurite branch points; migrating fibroblasts and astrocytes show AChE specifically at sites of membrane protrusion. The presence of AChE at neurite branch points indicates a possible role in stabilising adhesion of a potentially fragile region, or in the branching process itself [50].

### Interaction of AChE with laminin-1 reduces its catalytic activity

Unexpectedly for such a rapid enzyme as AChE, its active site is located at the bottom of a deep and narrow cleft, named the active-site gorge, lined by 14 conserved amino acid residues [51]. A second site, the PAS, surrounds the entrance to the active- site gorge, and is thought to play a role in the attraction and channeling of acetylcholine towards the active site [52]. The PAS has also been proposed to be involved in heterologous protein associations occurring during synaptogenesis or upon neurodegeneration [32], [53]. Two of the PAS residues, Tyr^72^ and Asp^74^, lie on a large omega loop; part of the loop forms a section of the wall of the active-site gorge, so that binding of the substrate on the AChE surface is able to influence the conformation of the gorge. Johnson and Moore [30] could demonstrate that laminin-1 binds to this surface loop adjacent to the peripheral anionic site. Binding of laminin-1 to this loop near to the entrance of the active site could affect the AChE activity by blocking the access of the substrate to the catalytic site. Laminin-1 is a large protein that simply by binding to AChE can obstruct the entrance of ACh into the gorge. However, if this is not the case, binding to this loop might cause conformational changes of the protein, which are shown to affect the catalytic activity of the enzyme. It was confirmed that ligand binding to PAS at the enzyme surface leads to a modulation of its catalytic activity [54], [55].

### AChE and laminin-1 together exert a more than additive effect on neurite growth

With this work we could confirm that the synaptic form of AChE including exon 6 has a neurite growth promoting function. We could also identify that the membrane- anchored form of AChE is more efficient in promoting neurite growth than the soluble secreted form. As expected, laminin-1 had also an effect in promoting neurite growth. But the novelty of this study is the fact that both molecules have together a more than additive effect on neurite growth. This suggests that the interaction of the two molecules is involved in regulation of neurite length in neural precursor cells.

There is clear evidence indicating a non-cholinergic involvement in fiber growth: De Jaco et al. [12] observed AChE-mediated neurite outgrowth in a cell line lacking acetylcholine, AChE inhibitors would not affect neurite growth in chick retinal neurons, but AChE overexpression will increase it [5]. Unfortunately, not much is known about the mechanism of AChE action. For example, it was demonstrated that exogenous application of acetylcholinesterase enhances neurite length by inducing an influx of calcium in a non-hydrolytic manner [56]. Another possible mechanism of regulation by micro RNAs (miRNAs) was reported. AChE expression can be regulated by different microRNAs, with one of the most prominent being miR132 [57]. miR132 is expressed in neurons, and is involved in processes of neurogenesis, regulating for example dendritic growth and arborisation in newborn neurons of adult hippocampus [58].

Laminin-1 itself controls neurite growth by interacting with integrins in both vivo and vitro [59]. A crucial role for integrin signaling was revealed by Gupton and Gertler [47]. Laminin activated integrin signaling which triggered the concomitant switch in cytoskeletal (FAK and src) and exocytic (Arp2/3 and VAMP7) machinery driving neuritogenesis [47]. It was postulated that laminin exerts a context-dependent influence over cell shape and behavior by inducing a coordinated switch in the cytoskeletal and exocytic machinery used to initiate neurite formation.

We may speculate that AChE binds to laminin-1, produces a signal that can enhance the affinity of laminin-1 for the α6β1 integrin receptor. The AChE binding site on laminin-1 is known for its role in mediating cell adhesion, neurite outgrowth and metastasis [60], [61].

Mouse AChE was observed to bind to a discontinuous, largely basic structure on the mouse laminin α1 G4 domain [61]. Other studies [23] showed that the binding site on laminin-1 is located on the N-terminal region of the β-chain and includes the G4 domain and a part of the cysteine-rich domain G3. Integrin binding to laminin requires three laminin globular domains, G1–3 in the alpha chains [62]. However, laminin alpha chain monomers do not show any significant activities for binding to integrins and require heterotrimerization with beta and gamma chains to fully exert their activities. The C-terminal region of laminin gamma chains is critically involved in laminin recognition by integrins [63]. It seems that both AChE and integrin bind to the same alpha chain of laminin, but to different globular domains. We can only speculate that the interaction of both molecules at the same time is structurally possible, or, if not, is a sequential one.

This study demonstrates that AChE-laminin interactions can affect neurite (out)growth tremendously, rendering their in vivo significance(s) highly likely.

## Materials and Methods

### Plasmids, proteins

The following plasmids were used during this study: pcDNA3-AChE mouse (includes part of exon 1 and exons 2, 3, 4, and 6), encoding the catalytic subunit of mouse tetrameric and asymmetric form of acetylcholinesterase, pcDNA3-AChE R395C [42] encodes a mutated form of AChE that leads to the retention of the protein within the cell (both plasmids were a generous gift of Dr. P. Taylor); pCMS-EGFP (Clontech, Germany) encodes the enhanced green fluorescent protein; pcDNA3-PRiMA [64] encoding the mouse PRiMA (proline-rich membrane anchor), encoding the acetylcholinesterase anchor in the mouse brain, which was a generous gift of Dr. Krejci.

Recombinant mouse AChE was purified on affinity columns from HEK293 cells overexpressing pcDNA3-AChE mouse with exon 6, and was a generous gift of Prof. Palmer Tayor. Alternatively, E6-AChE with a (his)_6_ tag was purchased from Sino Biological Inc. Laminin-1 was purchased from Sigma, Germany. A scheme of used plasmids is shown in Fig. 1C.

### Plasmid purification, RT-PCR

For transfection, plasmid DNA was purified using the alkaline lysis method. RNA was isolated using the RNeasy kit (Qiagen, Germany) or TRI-Reagent® (Sigma, Gemany), following the manufacturers protocol without modifications. 1 µg RNA per sample was used to generate cDNA using AMV-reverse transcriptase and oligo(dT)_15_ primer from Promega (Reverse Transcription Kit, Promega). Primers used to amplify were for GAPDH 5′ TGT TCC TAC CCC CAA TGT GT 3′, 5′ TGT GAG GGA GAT GCT CAG TG 3′ (396 bp); AChE mouse 5′ CAG CAA TAC GTG AGC CTG AA 3′, 5′ ATA CAG CTA GGG GCT CGG GC 3′ (414 bp); PRiMA mouse 5′ ACA AGC TTA TGC TAC TCC GG 3′, 5′ CAG AAT TCG CTCATG TCC AC 3′ (550 bp), integrin α6 5′ CGG GAA CTT CCT GAA AAA CA 3′, 5′ TTG TGG TAG GTG GCA TCG TA 3′ (464 bp), integrin ß1 5′ GAA CAG CAA GGG TGA AGC TC 3′, 5′ TTT CCA AAC CGT CAT GTG AA 3′(390 bp) and synthesized by Carl-Roth or Biomers.net (Germany). Cycle parameters were 1 min at 95°C, 1 min at 55°C, 1.5 min at 72°C; PCR was run for 30–35 cycles.

### Cell culture and transfections

R28, a rat retinal precursor cell line was generously provided by Dr. G. Seigel [41]. The cells were cultured in Dulbecco's Modified Eagle's Medium (DMEM, Gibco) supplemented with 10% fetal calf serum (FCS, Gibco), 1% L-glutamin, 20 units/ml penicillin and 20 µg/ml streptomycin at 37°C and 5% CO_2_. Stably transfected cells were cultured in the medium described above supplemented with 400 µg/ml G418 (geneticin, Sigma, Germany). The cells were seeded on 25 cm^2^ culture flasks or on glass cover slips. For laminin culturing, the flasks and coverslips were incubated for one hour with 5 µg/ml laminin-1 (Sigma) at 37°C. Parallel controls were run with flasks coated with poly-L-lysine or gelatin to avoid unspecific effects of the coating.

AChE, AChE R395C and PRiMA cDNAs were cloned in pcDNA3 which contains the neomycin gene under the control of the SV40 promoter for selection of stable transfectants. R28 cells were transfected at 60% confluence with 12 µg plasmid DNA, using Roti®fect and following the manufacturer protocol (Carl Roth, Germany). Neomycin resistant clones were selected by incubation for up to 1 month in the presence of 1 mg/ml G418, and isolated clones screened by cholinesterase activity test for elevated AChE. Stably transfected GFP clones were selected by control of the fluorescence and further subculturing of the green fluorescent colonies.

### Cell extracts and acetylcholinesterase activity measurements

Cells cultured in flasks were rinsed once with phosphate buffered saline and collected by mechanical dislodging. The cells were centrifuged and the proteins were extracted in Tris-HCl, pH7.5, containing 0.1% Triton X-100 and 1 µl/ml protease inhibitors (Protease inhibitor cocktail, Sigma). Cells were incubated 10 min on ice and sonicated for 2 times 30 seconds, followed by centrifugation for 10 min at 14000 rpm and 4°C. The supernatants were used for activity measurements.

Supernatants and media were collected and assayed for AChE activity using of 3 mM acetylthiocholine, plus 0.1 M Ellman buffer pH 8.0, and 0.6 mM DTNB in 500 µl final volume at 412 nm and 25°C [65]. Where iso-OMPA was used to inhibit activity, lysates were incubated in the presence of iso-OMPA for 6 min prior to substrate addition. OD was measured using a BioMate3 Spectrophotometer (Thermo Electronic, Germany) and activity was calculated with the help of the VISIONlite program Version 2.1 under the Quant Modus. Both media and cell-associated AChE activity were then normalized to the total cell protein content measured by Bradford [66]. All assays were carried out at least in triplicates. Possible interference of the lysis buffer and substrate autolysis was eliminated using different combinations of blank measurements.

### Immunohistochemistry, Karnovsky and Roots staining and microscopy

Parental R28 and AChE stably expressing cells were plated on glass coverslips, either not treated or coated with laminin-1and grown overnight in Dulbecco's modified Eagle's medium. Cells were fixed in 4% paraformaldehyde-PBS for 30 min at room temperature, washed and labeled for immunofluorescence. Briefly, anti-alpha tubulin monoclonal antibody (Sigma, Germany) was diluted at 1∶300 in phosphate buffered saline containing 5% bovine serum albumin (incubation for three hours at room temperature). Anti mouse (His)_6_ Tag antibody (Dianova) was diluted at 1∶100 in PBS or PBST and incubated for 3 h at room temperature. Cy2- or Cy3-conjugated rabbit anti-mouse secondary antibodies (Dianova, Hamburg) were diluted 1∶500 (incubation for one hour at room temperature). Finally, the sections were washed three times in PBS and the cell nuclei were stained with DAPI (0.1 µg/ml 4′,6-diamidine-2-phenylindol-dihydrochloride in PBS) for 1 min at room temperature. AChE histochemistry was used in order to follow the cholinesterase expression at the cellular level [67]. The glass coverslips were incubated for 10 min in 0.1 M Tris-maleate buffer, pH 6. After the equilibration step, the sections were incubated for up to 60 min in 0.1% acetylthiocholine, 0.1 M C_6_H_5_Na_3_O_7_X2H_2_O, 30 mM CuSO_4_, 5 mM K_3_Fe(CN)_6_ in Tris-maleate buffer. For cells transfected with AChE R395C, incubation time was extended to 6 hours.

The stainings were documented using a Zeiss Axiophot microscope with DIC (Nomarski) and fluorescence optics. Photomicrographs were taken using an Intas camera and a computer program (Diskus 1280, CH Hilgers, Königswinter). The figures were produced using Adobe Photoshop 7.

### Quantitative morphological analysis of neurite outgrowth and statistics

At the end of each incubation, cells plated on coverslips were fixed in 4% formaldehyde, permeabilized in 0.2% Triton X- 100, labeled with an anti-alpha-tubulin antibody (Sigma Aldrich, Germany) and mounted on microscope slides. The analysis was carried out blind; neurons whose processes were intermingled with those of neighboring cells were excluded from the analysis. Neurite length was measured from the point of emergence at the cell body to the tip of each segment. In each experiment, three coverslips per treatment were analyzed.

The total length of each neurite was calculated by Diskus 1280. Statistical analyses were performed with the aid of GraphPad Software. Values are presented as means ± standard error (SE) of at least triplicate experiments. Statistical analyses for all experiments were performed by one-way ANOVA, followed by Tukey HSD tests or by Students t-test. Values of p<0.05 were considered statistically significant.
